# Mini-Review: Regenerating the Corneal Epithelium With Simple Limbal Epithelial Transplantation

**DOI:** 10.3389/fmed.2021.673330

**Published:** 2021-05-28

**Authors:** Aastha Singh, Virender S. Sangwan

**Affiliations:** Department of Cornea, Anterior Segment and Uveitis, Dr. Shroff's Charity Eye Hospital, New Delhi, India

**Keywords:** limbal stem cells, limbal stem cell deficiency, limbal stem cell transplantation, simple limbal epithelial transplantation, corneal regeneration

## Abstract

Simple limbal epithelial transplantation (SLET) is an ingenious, low cost and effective technique of limbal stem cell transplantation (LSCT) that is increasingly being undertaken in practice across the world. Since it was first described a decade ago, the technique has been performed in a variety of cases of limbal stem cell deficiency (LSCD) and has underwent several innovative modifications. Published literature on SLET has progressively increased over time and successful outcomes in various clinical scenarios have been reported. This concise review attempts to present a crisp account of SLET covering the indications and contraindications of performing the procedure; detailed account of pre-operative work up and preparation; surgical technique and its modifications; post-operative course, care and possible complications as well as published outcomes of surgery from across the world. Comparative analysis of various techniques of LSCT have been discussed and common concerns of surgeons practising or those who are planning to start practising SLET have been addressed. The authors hope that the pragmatic insights and pearls given at the end of the review will aid the surgeons in performing this technique to provide maximum benefit to patients suffering from the potentially blinding condition of LSCD.

## Introduction

Simple limbal epithelial transplantation (SLET) is a relatively new technique of limbal stem cell transplantation first described by Sangwan et al. in the year 2012 (1). After having worked with the technique of cultivated limbal epithelial transplantation (CLET) for close to a decade, Sangwan et al. devised this unique and low cost technique of stem cell transplantation while working on an alternative to the human amniotic membrane (hAM) (2, 3).

The cornea is covered by non-keratinised, stratified squamous epithelium which undergoes constant renewal by limbal stem cells (LSCs). These cells are located in their niche in the radially oriented palisades of Vogt and limbal epithelial crypts (4). The LSCs are a population of adult stem cells that drive the regeneration of the corneal surface. They give rise to the transient amplifying cells (TACs) which migrate centripetally along the basement membrane to replace the epithelial cells during normal hameostasis or following injury. The TACs first differentiate to post-mitotic cells of supra basal corneal epithelium and finally differentiate in to terminal differentiated cells (5).

Damage to limbal stem cells or their niche results in a condition termed limbal stem cell deficiency (LSCD) which is clinically characterised by conjunctivalisation, neovascularisation, persistent epithelial defect and inflammation of the ocular surface. The corneal surface in such a scenario undergoes frequent epithelial breakdown and has a poor healing potential leading to melting and scarring. LSCD thus is a potentially blinding condition. LSCD can affect either one or both the eyes and can be either partial or total. Broadly, trauma, inflammation and congenital morbidities are the main causes of LSCD. Primary LSCD is seen in conditions such as aniridia, epidermal dysplasias, congenital erythrokeratodermia and dyskeratosis congenita while secondary causes of LSCD include chemical or thermal burns, inflammatory eye diseases such as vernal keratoconjunctivitis (VKC), Stevens-Johnson syndrome(SJS), ocular cicatricial pemphigoid (OCP), pseudo pemphigoid, ocular surface squamous neoplasia (OSSN), pterygium and neurotrophic keratitis (6).

Persistent epithelial defects due to LSCD are managed by clinicians with bandage contact lens (BCL), tarsorrhaphy, amniotic membrane transplantation(AMT) or by various techniques of limbal stem cell transplantation (LSCT) (7–9). LSCT involves transplanting of limbal stem cells along with their niche to the recipient eye and it thus addresses the underlying pathophysiology directly. The technique of LSCT has undergone various refinements since it was first proposed by Jose Barraquer in 1964 for treating autologous superficial burns. Extensive research on the basic science behind epithelial regeneration has allowed the clinicians to modify, improvise and simplify the technique for obtaining better outcomes and expand its outreach. Commonly practised techniques of LSCT today are conjunctival limbal autograft (CLAU), CLET or SLET. This concise review aims to present the surgical technique of SLET, its modifications, outcomes and insights to improve the success rate of the procedure.

## Goals of Treatment, Indications, and Contraindications of SLET

The primary goal of therapy with SLET is to establish a well-epithelised corneal surface in cases of LSCD. Regeneration of epithelium of the corneal phenotype is of utmost importance for globe salvage as well as visual rehabilitation of these patients. Keratoplasty alone in these patients is not enough as only the central part of cornea is grafted in keratoplasty leaving the LSCD unaddressed. This results in poor epithelisation of these grafts leading to secondary keratitis, melting, growth of pannus on to the graft and eventual failure of the procedure (10, 11).

**Figure 1:**
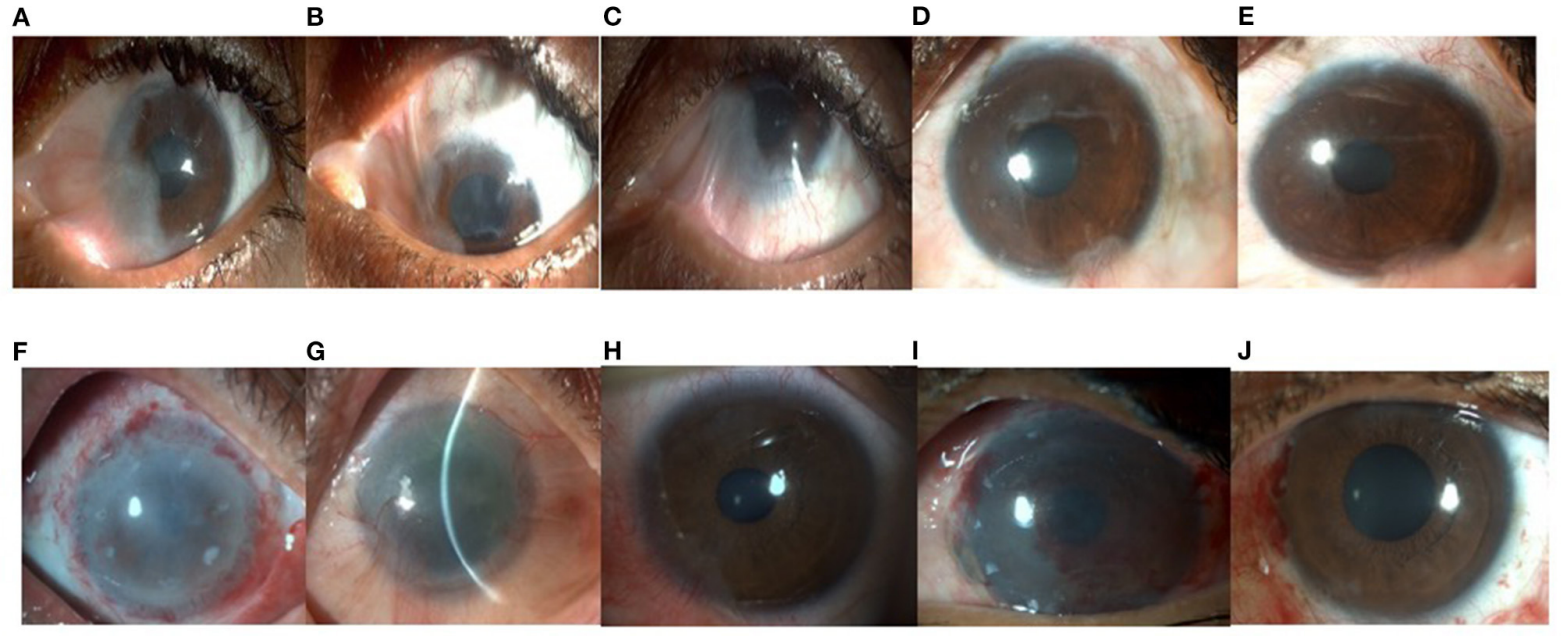
**(A)** Clinical photograph of a 37 year old lady who developed partial limbal stem cell deficiency(LSCD) involving 6 clock hours of the limbus with **(B)** superior and, **(C)** inferior symblepharon in the left eye following trauma with bleaching powder. Best corrected visual acuity (BCVA) in the affected (left) eye was 20/200. She underwent symblepharon release and autologous simple limbal epithelial transplantation(SLET) in the left eye. **(D)** At post-operative 2 months, the patient had a well epithelised ocular surface and best corrected visual acuity of 20/30. **(E)** At post-operative 6 months, ocular surface was healthy, explants were seen to be fading and the patient's BCVA was 20/20. **(F)** A 54 year old gentleman underwent SLET for total LSCD in the right eye following accidental injury with acid (household cleaning agent). **(G)** One year post operatively his BCVA was 20/30 with significant clearing of the visual axis. **(H)** Clinical photograph of a 24 year old boy who presented with partial LSCD following chemical injury. **(I)** On post-operative day 1, limbal stem cell explants can be seen well placed over the human amniotic membrane (hAM). **(J)** At post-operative 3 months, patient had a well epithelised, clear cornea and stable ocular surface.

SLET has shown promising results in treating LSCD due to chemical/thermal injury (Figure 1), allergic eye diseases, pterygium, OSSN, failed prior CLET and some cases of burnt out VKC, SJS, and OCP (12). However, caution is warranted in cases with concomitant lid abnormalities, severe dryness of the ocular surface, extensive conjunctival scarring, symblepheron and acute inflammation of the eye. The aforementioned scenarios are relative contraindications of carrying out the procedure. Presence of stromal thinning, stromal opacification, raised intraocular pressure, previous failed penetrating keratoplasty or LSCT, history of multiple surgeries and undertaking concomitant keratoplasty are other poor prognostic factors.

## Surgical Technique

Timing of surgery is crucial to the success of SLET. It should be undertaken in chronic stage of the disease where in most of the inflammation of the eye has subsided. In cases of ocular burns, this could be 12–18 months post-injury. Anti-inflammatory medications (topical or systemic) are prescribed and ocular surface inflammation must be well-controlled before scheduling surgery. Co-existing lid margin keratinisation, must be addressed with mucous membrane grafting at least 3 months prior. Other adnexal pathologies such as entropion, ectropion, trichiasis or dacryocystitis must be treated before undertaking SLET. Ocular surface must be relatively wet and procedures such as punctal occlusion if required must be performed to restore sufficient moisture.

The two critical steps of performing SLET are host bed preparation and harvesting of donor stem cells. A healthy, undisturbed donor site with viable tissue is critical for a successful outcome. Typically, the superior limbus is the preferred site for donor tissue harvesting as the limbal palisades are more in number at this location. In cases of cadaveric SLET, fresh tissue, ideally harvested <48 hours prior to surgery with visible intact limbal palisades, healthy epithelium and preferably from a donor <60 years of age must be selected.

SLET can be performed either under peribulbar or general anaesthesia depending on the age and systemic condition of the patient. For healthy adults, local anaesthesia is sufficient for performing surgery. It is recommended to administer brimonidine tartrate 0.15% eye drops two to three times in both donor and recipient eyes 10 min prior to surgery. This acts as a chemical cautery and minimises per operative bleeding.

In cases of autologous SLET, both eyes are anesthetised and prepared. One clock hour of limbal biopsy (3−4 mm) from the contralateral healthy superior limbus is dissected. In cases of partial LSCD, limbal biopsy can be harvested from the same eye. A limbal based conjunctival flap is raised and sub-conjunctival dissection is carried out until the limbus is reached. A shallow dissection is then performed 1 mm into the clear cornea using a 15 No. surgical blade on a Bard Parker (BP) handle. The donor tissue is excised from the base using a Vannas scissor and placed in balanced salt solution. It is then kept aside safely on the surgical trolley. This is followed by preparation of the recipient bed.

Firstly, symblephera if any are released and the globe is rendered fully mobile. Conjunctival peritomy is then done 4–5 mm beyond the limbus and bleeders are cauterised. Pannus dissection is a crucial step and meticulous dissection is advised. The pannus is completely dissected off the cornea using a 15 No surgical blade on a BP handle. Appropriate plane with the grey cornea beneath is identified and dissection is carried out placing the blade as flat as possible to the surface. Care must be taken to avoid deep dissection especially in suspected areas of thinning.

Human amniotic membrane (hAM) with its epithelial side up, is then glued to the bed with fibrin glue (TISSEEL Kit from Baxter AG, Vienna, Austria). It is firmly tucked under the conjunctiva and smoothened out. The harvested donor tissue is divided into 10–12 smaller pieces with Vannas scissors and the explants with their epithelial side up are then placed on top of the secured amniotic membrane in a circular fashion starting from mid periphery while avoiding the visual axis. A bandage contact lens (BCL) is placed at the end of the procedure.

## Post-operative Care, Follow Up, Course, and Complications

### Post-operative Treatment

Post-operatively, broad spectrum preservative free antibiotic eyedrop such as moxifloxacin (0.5%) eyedrop is prescribed four times a day until healing of the epithelial defects. Topical prednisolone acetate 1% eye drop is administered six times a day for a week and then tapered weekly over the next 6 weeks in both the recipient and the donor eyes. Topical corticosteroids are recommended to be continued for a longer period on low maintenance dose in the recipient eye. Carboxy methyl cellulose 0.5% can be prescribed four times a day for both the recipient and donor eye. In cases of allogenic SLET systemic immune suppression is prescribed as per established regimes (13).

### Follow Up

In the immediate post-operative period, patients are kept under a close follow-up until the epithelium heals. It is recommended to follow up the patients on Day 1, Day 7 and thereafter at weeks 2, 6, and 12 post surgery. They can be followed up at 3 monthly interval thereafter.

On subsequent follow ups, the following parameters namely epithelisation status, recurrence of LSCD, clarity of the cornea, retention and size of the explants, signs of rejection (in cases of allograft) as well as health of the donor site must be noted. If the patient is on immune suppression, dosage of the same is monitored and tapered over time. Monitoring of blood counts, blood sugar levels, liver and kidney function tests are essential in these patients.

### Course

Bandage contact lens is removed at 1 week to check the epithelisation status and can be safely removed once the epithelisation is complete. Sangwan et al. had noted in their study that the fibrin glue had disintegrated by the end of the first post-operative week, and the hAM disintegrated gradually over 6 weeks in their patients. The donor site had epithelised by day 14 in all eyes and none of the donor eyes had showed development of iatrogenic LSCD. The transplanted stem cells became progressively transparent over time and completely disappeared by 6 months in their study (1).

In an *in vivo* study, the first clinical evidence of proliferation of the corneal epithelium from the limbal explants was seen on the second day and ocular surface epithelialisation was complete in all cases within 14 days (14). Variations were seen in explant activity with the size and age of the explants and pattern of ocular surface epithelialisation was similar to that observed in CLET (14).

Additionally, anterior segment optical coherence tomography (AS- OCT) has been used as a tool to trace epithelial regeneration post SLET. Amescua et al. have described the growth of epithelial sheet over the hAM post SLET using OCT (15). Chaudhury et al. have meticulously demonstrated on OCT that the hAM is intact and of the same thickness while the sub-hAM space increases from day 3 to 9 following SLET (16). They reported that epithelialisation occurred between day 8 and 14 and proceeded more rapidly towards the limbus than centrally (16). The explants showed to start thinning from day 3 with the fibrin around them starting to decrease from day 2 and completely disappearing by day 4 in their report (16).

### Complications

Rare complications to the donor eye include subconjunctival haemorrhage, pyogenic granuloma formation, or iatrogenic LCSD. Complications in the recipient eye include corneal perforation at the time of pannus dissection, post-operative keratitis, detached amniotic membrane and loss of limbal transplants. Failure of the procedure is most often attributable to superficial plane of dissection during graft harvesting leading to inadequate quantity of stem cells. Rough handling and improper orientation while placing the explants can further compromise success of the procedure.

## Modifications of SLET

True to its nature of being an innovative technique, SLET has undergone several modifications by clinicians across the globe. Amescua et al. in 2014, described the use of a double-layer cryopreserved amniotic membrane with limbal transplants being sandwiched within the two layers (15). Vazirani et al. have reported “customised SLET” for treating multiple focal patches of LSCD and concomitant primary SLET after surgical excision of OSSN has been reported by Kaliki et al to prevent inevitable LSCD arising after extensive dissection in such cases (17, 18).

Minor ipsilateral SLET (mini-SLET) has been described for the treatment of pterygium by Hernandez-Bongates et al. It was reported to provide benefits of less tissue requirement and lower chances of recurrence than conventional conjunctival autograft technique for treating pterygium (19). This has been further proved in a randomised clinical trial by Sati et al. who demonstrated a positive trend of less recurrence with reduced post-operative symptoms with mini-SLET for the management of pterygium (20). Recently, glue less SLET (G-SLET) has been described by Malyugin et al. In this modification, limbal explants were inserted in surgically created short oblique or horizontal tunnels in the corneal periphery followed by placement of amniotic membrane on the top. This can be particularly useful for settings in which fibrin glue may not be available (21).

## Analysis of Existing Evidence on SLET in Regenerating the Human Corneal Epithelium

The pioneering work on SLET by Sangwan et al. reported successful primary outcome of the procedure defined as a completely epithelialised, avascular and stable corneal surface at 6 weeks in all of their 6 patients. This was maintained at a mean follow up of 9.2 ± 1.9 months. Best corrected visual acuity (BCVA) improved from worse than 20/200 before surgery to 20/40 or better in four (66.6%) eyes in their study (1).

The largest series reported on SLET is by Basu et al. (125 cases). In their study, at a median post-operative follow-up of 1.5 years, 76% of the patients maintained a successful outcome with two-line improvement in visual acuity seen in 75.2% of the patients, and 67% of successful cases attained 20/60 or better vision. Results were particularly promising in paediatric patients with 71.2% of children with total LSCD showing complete epithelisation following SLET. Encouraging results in paediatric patients were also reported by Gupta et al. who reported anatomical success in 80% and functional success in 85.7% of paediatric patients receiving SLET (22). A multicentre centre study comprising of patients from eight centres in three countries reports successful outcomes in 83.8% of the patients with 65% of patients achieving visual acuity of 20/200 or better (17). SLET has also showed successful results in cases of previously failed CLET and has shown to have better outcomes than a repeat CLET in such patients (23).

Several published case reports describe the success of SLET in various clinical scenarios such as for treatment of pterygium, ocular surface neoplasia, conjunctival melanoma and acid injury with dry eye (24–26). Modifications of SLET such as sandwich technique, G-SLET and mini SLET have all showed promising results (15, 19, 21).

The outcomes of sequential secondary surgeries such as penetrating keratoplasty (PK) and deep anterior lamellar keratoplasty (DALK) after SLET have also been reported in literature. Gupta et al. have reported maintenance of clear graft at 15 months post-PK in eyes previously treated with SLET in 85% of patients (11). Similarly Singh et al. reported anatomical success in 72% of the eyes and visual acuity improvement in 54% of the eyes undergoing DALK post-SLET (27).

## Comparative Analysis of Limbal Stem Cell Transplantation Procedures

LSCT can be performed by primarily three techniques namely conjunctival-limbal autografting (CLAU), first described by Kenyon and Tseng in 1989, cultivated limbal epithelial transplantation (CLET) described by Pellegrini et al. in 1997 and SLET (28, 29). CLAU comprises of transplanting large conjunctival-limbal grafts and is frequently associated with the risk of developing iatrogenic LSCD in the donor eye. As compared to SLET or CLET where multidirectional epithelial growth from explants leads to faster healing, in CLAU there is unidirectional epithelial growth and central cornea is the last to heal (14).

In CLET, a tiny limbal biopsy from the healthy eye is cultured in a laboratory to create a multi layered sheet of corneal epithelium which is transplanted in the patient in a second surgery. The major limitations of this technique are, it being a two staged procedure and the need of a clinical-grade laboratory which requires regulatory approval and trained manpower that increases the cost to manifold. SLET however in comparison is a low cost procedure in which the patient's own eye is used as an incubator for corneal epithelial regeneration. Cost analysis suggests that SLET can be performed at one tenth of the cost of CLET (30). Its added advantages being repeatability and superior outcomes in paediatric patients as compared to CLET (31, 32).

Parameters and performance of these three techniques are further discussed in Table 1.

**Table 1:** Comparison of various limbal stem cell transplantation (LSCT) techniques.

	**Parameter**	**Comparison**
1	Donor tissue size	SLET= CLET >> CLAU
2	Donor eye safety	SLET =CLET > CLAU
3	Epithelisation pattern	SLET=CLET > CLAU
4	Success rates	SLET = CLET > CLAU (Adults)SLET > CLET > CLAU (Paediatric patients)
5	Visual outcomes	SLET = CLAU > CLET
6	Affordability	SLET = CLAU >> CLET
7	Repeatability	SLET > CLET >> CLAU

*SLET, Simple limbal epithelial transplantation; CLET, Cultivated limbal epithelial transplantation; CLAU, Conjunctival limbal autograft*.

## Insights and Pearls for Performing SLET

Choice of anaesthesia is a frequent concern with surgeons who are starting out with performing SLET. Local anaesthesia is sufficient for undertaking the procedure however in paediatric and anxious patients general anaesthesia is preferable. Another concern is regarding the sequence of surgical steps. The authors harvest limbal biopsy prior to pannus dissection and recommend the same. In the case of accidental perforation while pannus dissection, it can be sealed with either cyanoacrylate glue or a tenon patch graft and SLET is deferred.

Depth of dissection while harvesting limbal biopsy is crucial to maximise outcome of the surgery. If the dissection is superficial, it can lead to inadequate limbal stem cells and their niche and failure of the procedure is inevitable. Dissection under direct visualisation up to 1 mm into the clear cornea, in the plane of the insertion of the tenons capsule to the limbus is recommended. For pannus dissection we recommend finding the plane of dissection flush to the limbus and moving inwards. A 15 No surgical blade on BP handle along with sharp vannas scissors can be used to carry out meticulous dissection till clear corneal surface is visible underneath. Care must be taken to identify areas of thinning to avoid iatrogenic perforation. A pre-operative AS-OCT is often useful in delineating areas of thinning and can forewarn the surgeon.

A well fitting BCL should be placed at the end of surgery and a tarsorrhaphy is performed in cases where retention of BCL seems doubtful in the post-operative period such as in paediatric patients.

Another query among surgeons is if SLET can be combined with lamellar or full thickness keratoplasty. In cases of obvious volume loss a deep anterior lamellar keratoplasty (DALK) or penetrating keratoplasty (PK) is recommended along with SLET. In patients with a failed PK with LSCD, either SLET can be performed first followed by a repeat PK at a later date or a combined surgery can be done at the same setting.

In all cases, informed consent should be taken and expected realistic outcome of the procedure, post-operative course, need for multiple surgeries as well as goal of surgery should be well communicated to the patient or guardian. A thorough pre-operative work up including assessment of visual potential and co morbidities must be done. Additionally, it is expected that the surgeon undertaking SLET should be familiar with performing ocular surface surgeries and handling of fibrin glue. The surgeon must be aware of the adverse post-operative events and adept in taking suitable course of action in case of such events.

## Concluding Remarks

SLET is an effective procedure to restore the corneal surface and improve vision in patients with LSCD. It requires minimal amount of tissue to regenerate the corneal epithelium. It is a repeatable and reliable procedure that has been shown to work equally well in children as well as in adults. Replicability of SLET has been remarkable and various groups across the world have implemented the procedure as well as introduced varied modifications with consistent good results. True to its name, it is a fairly straightforward technique which is easy to grasp and reproduce even by surgeons with lesser experience.

SLET negates the requirement of a sophisticated laboratory that requires trained manpower and strict regulatory control. This makes it accessible to more number of surgeons who do not have access to a specialised laboratory required for cell-based technique. Being accessible to more number of surgeons, makes the therapy accessible in turn to more number of patients. Risk of contaminations associated with *ex vivo* tissue expansion is additionally circumvented. As SLET is a single stage surgery it is both convenient and economical for the patients.

While developing the technique of SLET, the authors had hoped for a technology which was uncomplicated, effective and could have a widespread reach. A decade later, SLET continues to serve these goals while adapting and expanding its realm.

## Author Contributions

Conception, design, editing, and reviewing of the manuscript have been done by VS. Review of literature, data collection, and analysis have been done by AS. Both authors have contributed to writing the manuscript, providing critical feedback and in compiling and shaping of the draft. The manuscript has the final approval of both authors.

## Conflict of Interest

The authors declare that the research was conducted in the absence of any commercial or financial relationships that could be construed as a potential conflict of interest.

## Abbreviations

SLET: simple limbal epithelial transplantation
LSCT: limbal stem cell transplantation
LSCD: limbal stem cell deficiency
VKC: vernal keratoconjunctivitis
SJS: Stevens-Johnson syndrome
OCP: ocular cicatricial pemphigoid
OSSN: ocular surface squamous neoplasia
CLAU: conjunctival limbal autograft
CLET: cultivated limbal epithelial transplantation
hAM: human amniotic membrane
BCL: bandage contact lens
AS-OCT: anterior segment optical coherence tomography.

## References

[1] Sangwan VS Basu S MacNeil S Balasubramanian D. Simple limbal epithelial transplantation (SLET): a novel surgical technique for the treatment of unilateral limbal stem cell deficiency. Br J Ophthalmol. (2012) 96:931–4. 10.1136/bjophthalmol-2011-301164 22328817

[2] Sangwan VS Basu S Vemuganti GK Sejpal K Subramaniam SV Bandyopadhyay S. Clinical outcomes of xeno-free autologous cultivated limbal epithelial transplantation: a 10-year study. Br J Ophthalmol. (2011) 95:1525–9. 10.1136/bjophthalmol-2011-300352 21890785

[3] Sangwan VS Gupta N Singh A MacNeil S. Cutting corners, or simplifying technology to reach more patients; using the body as its own incubator for epithelial regeneration. Indian J Ophthalmol. (2019) 67:1261–3. 10.4103/ijo.IJO_632_19 31332104 PMC6677077

[4] Dua HS Azuara-Blanco A. Limbal stem cells of the corneal epithelium. Surv Ophthalmol. (2000) 44:415–25. 10.1016/S0039-6257(00)00109-0 10734241

[5] Dua HS. Stem cells of the ocular surface: scientific principles and clinical applications. Br J Ophthalmol. (1995) 79:968–9. 10.1136/bjo.79.11.968 8534664 PMC505307

[6] Vazirani J Nair D Shanbhag S Wurity S Ranjan A Sangwan V. Limbal stem cell deficiency—demography and underlying causes. Am J Ophthalmol. (2018) 188:99–103. 10.1016/j.ajo.2018.01.020 29378178

[7] Kheirkhah A Casas V Raju VK Tseng SCG. Sutureless amniotic membrane transplantation for partial limbal stem cell deficiency. Am J Ophthalmol. (2008) 145:787–94. 10.1016/j.ajo.2008.01.009 18329626 PMC2840647

[8] Singh A Murthy SI Gandhi A Sangwan VS. “Doughnut” amniotic membrane transplantation with penetrating keratoplasty for vernal keratoconjunctivitis with limbal stem cell disease. Cornea. (2020). 10.1097/ICO.0000000000002553. [Epub ahead of print]. 33214419

[9] Acharya M Gour A Dave A. Commentary: tarsorrhaphy: a stitch in time. Indian J Ophthalmol. (2020) 68:33–4. 10.4103/ijo.IJO_1824_19 31856461 PMC6951190

[10] Basu S Mohamed A Chaurasia S Sejpal K Vemuganti GK Sangwan VS. Clinical outcomes of penetrating keratoplasty after autologous cultivated limbal epithelial transplantation for ocular surface burns. Am J Ophthalmol. (2011) 152:917–24.e1. 10.1016/j.ajo.2011.05.019 21851920

[11] Gupta N Farooqui JH Patel N Mathur U. Early results of penetrating keratoplasty in patients with unilateral chemical injury after simple limbal epithelial transplantation. Cornea. (2018) 37:1249–54. 10.1097/ICO.0000000000001681 29975208

[12] Shanbhag SS Patel CN Goyal R Donthineni PR Singh V Basu S. Simple limbal epithelial transplantation (SLET): review of indications, surgical technique, mechanism, outcomes, limitations, and impact. Indian J Ophthalmol. (2019) 67:1265–77. 10.4103/ijo.IJO_117_19 31332106 PMC6677059

[13] Serna-Ojeda JC Basu S Vazirani J Garfias Y Sangwan VS. Systemic immunosuppression for limbal allograft and allogenic limbal epithelial cell transplantation. Med Hypothesis Discov Innov Ophthalmol. (2020) 9:23–32. 31976340 PMC6969562

[14] Mittal V Jain R Mittal R. Ocular surface epithelialization pattern after simple limbal epithelial transplantation: an *in vivo* observational study. Cornea. (2015) 34:1227–32. 10.1097/ICO.0000000000000573 26266437

[15] Amescua G Atallah M Nikpoor N Galor A Perez VL. Modified simple limbal epithelial transplantation using cryopreserved amniotic membrane for unilateral limbal stem cell deficiency. Am J Ophthalmol. (2014) 158:469–75.e2. 10.1016/j.ajo.2014.06.002 24932987

[16] Ray Chaudhuri B Bhaduri A Sengupta M. The ocular surface after simple limbal epithelial transplant (SLET): a high-resolution OCT study of the early postoperative period. Indian J Ophthalmol. (2019) 67:1348–50. 10.4103/ijo.IJO_1722_18 31332139 PMC6677049

[17] Vazirani J Lal I Sangwan V. Customised simple limbal epithelial transplantation for recurrent limbal stem cell deficiency. BMJ Case Rep. (2015) 2015:bcr2015209429. 10.1136/bcr-2015-209429 26082100 PMC4480128

[18] Kaliki S Mohammad FA Tahiliani P Sangwan VS. Concomitant simple limbal epithelial transplantation after surgical excision of ocular surface squamous neoplasia. Am J Ophthalmol. (2017) 174:68–75. 10.1016/j.ajo.2016.10.021 27832940

[19] Hernández-Bogantes E Amescua G Navas A Garfias Y Ramirez-Miranda A Lichtinger A. Minor ipsilateral simple limbal epithelial transplantation (mini-SLET) for pterygium treatment. Br J Ophthalmol. (2015) 99:1598–600. 10.1136/bjophthalmol-2015-306857 26130669 PMC4680150

[20] Sati A Banerjee S Kumar P Kaushik J Khera A. Mini-simple limbal epithelial transplantation versus conjunctival autograft fixation with fibrin glue after pterygium excision: a randomized controlled trial. Cornea. (2019) 38:1345–50. 10.1097/ICO.0000000000002007 31436643

[21] Malyugin BE Gerasimov MY Borzenok SA. Glueless simple limbal epithelial transplantation: the report of the first 2 cases. Cornea. (2020) 39:1588–91. 10.1097/ICO.0000000000002467 32925431

[22] Gupta N Joshi J Farooqui JH Mathur U. Results of simple limbal epithelial transplantation in unilateral ocular surface burn. Indian J Ophthalmol. (2018) 66:45–52. 10.4103/ijo.IJO_602_17 29283122 PMC5778581

[23] Basu S Mohan S Bhalekar S Singh V Sangwan V. Simple limbal epithelial transplantation (SLET) in failed cultivated limbal epithelial transplantation (CLET) for unilateral chronic ocular burns. Br J Ophthalmol. (2018) 102:1640–5. 10.1136/bjophthalmol-2017-311506 29453224

[24] Mednick Z Boutin T Einan-Lifshitz A Sorkin N Slomovic A. Simple limbal epithelial transplantation for recurrent pterygium: a case series. Am J Ophthalmol Case Rep. (2018) 12:5–8. 10.1016/j.ajoc.2018.07.006 30101206 PMC6083897

[25] Boutin T Mednick Z Zhou TE Showail M Einan-Lifshitz A Sorkin N. Simple limbal epithelial transplantation to treat recurring kissing pterygium. Can J Ophthalmol J Can Ophtalmol. (2019) 54:e54–7. 10.1016/j.jcjo.2018.06.003 30975360

[26] Arya SK Bhatti A Raj A Bamotra RK. Simple limbal epithelial transplantation in acid injury and severe dry eye. J Clin Diagn Res. (2016) 10:ND06–07. 10.7860/JCDR/2016/19306.7997 27504323 PMC4963683

[27] Singh D Vanathi M Gupta C Gupta N Tandon R. Outcomes of deep anterior lamellar keratoplasty following autologous simple limbal epithelial transplant in pediatric unilateral severe chemical injury. Indian J Ophthalmol. (2017) 65:217–22. 10.4103/ijo.IJO_880_16 28440250 PMC5426126

[28] Kenyon KR Tseng SC. Limbal autograft transplantation for ocular surface disorders. Ophthalmology. (1989) 96:709–22. Discussion 722–3. 10.1016/S0161-6420(89)32833-8 2748125

[29] Pellegrini G Traverso CE Franzi AT Zingirian M Cancedda R De Luca M. Long-term restoration of damaged corneal surfaces with autologous cultivated corneal epithelium. Lancet Lond Engl. (1997) 349:990–3. 10.1016/S0140-6736(96)11188-0 9100626

[30] Thokala P Singh A Singh VK Rathi VM Basu S Singh V. Economic, clinical and social impact of simple limbal epithelial transplantation for limbal stem cell deficiency. Br J Ophthalmol. (2021) 9. 10.1136/bjophthalmol-2020-318642 33688000 PMC9234414

[31] Sejpal K Ali MH Maddileti S Basu S Ramappa M Kekunnaya R. Cultivated limbal epithelial transplantation in children with ocular surface burns. JAMA Ophthalmol. (2013) 131:731–6. 10.1001/jamaophthalmol.2013.2308 23559315

[32] Ganger A Vanathi M Mohanty S Tandon R. Long-term outcomes of cultivated limbal epithelial transplantation: evaluation and comparison of results in children and adults. BioMed Res Int. (2015) 2015:480983. 10.1155/2015/480983 26770973 PMC4681831

